# Overweight and Obesity as Risk Factors for Recurrent Herpetic Stromal Keratitis during Long-Term Antiviral Prophylaxis

**DOI:** 10.3390/v14122812

**Published:** 2022-12-16

**Authors:** Chiung-Ju Hsu, Jia-Horung Hung, I-Huang Lin, Sung-Huei Tseng, Sheng-Hsiang Lin, Yi-Hsun Huang

**Affiliations:** 1 Department of Ophthalmology, National Cheng Kung University Hospital, College of Medicine, National Cheng Kung University, Tainan 704, Taiwan; 2 Institute of Clinical Medicine, College of Medicine, National Cheng Kung University, Tainan 704, Taiwan; 3 Department of Public Health, College of Medicine, National Cheng Kung University, Tainan 704, Taiwan; 4 Biostatistics Consulting Center, National Cheng Kung University Hospital, College of Medicine, National Cheng Kung University, Tainan 704, Taiwan

**Keywords:** body mass index, herpetic keratitis, obesity, overweight

## Abstract

Although past research has shown an association between obesity and herpes simplex virus infection, the relationship between body mass index (BMI) and herpetic stromal keratitis (HSK) recurrence has never been investigated. In this study, we included HSK patients who received oral valacyclovir as prophylactic treatment between January 2016 and January 2021. Recurrence, possible risk factors, and the time to recurrence were recorded during follow-ups. Among the 56 patients included in this study, recurrence was reported in 21 (37.5%) patients. The age at disease onset and mean follow-up time were not significantly different in the recurrence and non-recurrence groups. However, in the Cox regression analysis, BMI ≥ 24 kg/m^2^ was noted as the variable having significant correlation with recurrence (*p* = 0.01 in univariate analysis and *p* = 0.001 in multivariate analysis). In conclusion, overweight and obesity were revealed as risk factors for HSK recurrence in patients receiving long-term antiviral prophylaxis. Further studies are needed to determine the appropriate acyclovir concentrations in the blood or aqueous humour in order to achieve desirable prophylactic effects, especially in the overweight and obese patients.

## 1. Introduction

Herpetic keratitis, caused by herpes simplex virus 1 (HSV-1) infection, involves both anterior and posterior eye segments and results in 40,000 new annual cases of visual impairment or blindness worldwide [1]. The prevalence of herpetic keratitis ranges from 31.5 to 149 per 100,000 population with a high recurrence rate of up to 18.3 per 100,000 person-years [2,3]. Herpetic stromal keratitis (HSK) is thought to result from T-cell-mediated inflammatory response to the virus, and to be the most common type of herpetic eye disease (HED). Non-necrotizing HSK presents with localized stromal edema, while necrotizing HSK presents with suppurative stromal infiltrates and inflammation. Vision impairment is associated with stromal scarring or stromal neovascularization [1]. For the past 30 years, clinical treatment guidelines for herpetic keratitis have been based on the HED study (HEDS), which suggests a 12-month oral acyclovir (ACV) treatment regimen to reduce the recurrence of HSK [4,5,6,7].

ACV is an antiviral drug that targets HSV-infected cells. It inhibits replication and shedding of HSV by binding to the viral DNA polymerase [8]. ACV has been used widely to treat and prevent the recurrence of genital, orofacial, and ocular herpes [9,10]. Valacyclovir (VACV) is another antiviral drug which has a three to five times higher bioavailability than ACV [8]. It has been demonstrated that prophylactic treatment with oral VACV (500 mg tablet daily) is as effective as ACV (400 mg tablet twice daily) in reducing recurrent HSK without severe adverse events [11]. Therefore, oral VACV has emerged as one of the suggested prophylactic treatment options for HSK in recent times [12,13]. Despite the availability of antiviral agents that treat and prevent recurrent HSK, the recurrence rate still remains as high as 19.0% to 36.4% under prophylactic antiviral treatment [7,11,14]. Studies in the past have suspected fever, hormonal changes, ultraviolet exposure, psychological stress, ocular trauma, and trigeminal nerve manipulation as risk factors for recurrence of HSV, but no strong associations have been found [15,16,17].

Intriguingly, it has been proposed that there is a reciprocal association between obesity and microbial infections [18]. A previous study has shown that HSV modulated adipocyte function by enhancing adipose cell proliferation and reducing leptin release, indicating that some innate antiviral responses induced by HSV may contribute to adipogenesis and obesity [19]. In addition, Karjala et al. reported that the prevalence of HSV infection in patients with obesity was over 60%, and significantly higher than that in the normal-weight patients [20]. Moreover, higher body mass index (BMI) is thought to be a risk factor for numerous eye diseases, including keratoconus, age-related cataracts, elevated intraocular pressure, age-related macular degeneration, and diabetic retinopathy [21,22]. However, the association between BMI and HSK recurrence has never been investigated. Therefore, in this study we aimed to evaluate the clinical outcomes and the risk factors of recurrent HSK in patients on antiviral prophylaxis. Furthermore, we investigated the impact of BMI on HSK recurrence during long-term antiviral prophylaxis.

## 2. Materials and Methods

### 2.1. Patient Selection

This study was approved by the Institutional Review Board (approval no. B-ER-110-238, 3August 2021) of the National Cheng Kung University Hospital (NCKUH), Taiwan. We retrospectively reviewed electronic medical records of newly diagnosed HSK patients who received oral VACV treatment from January 2016 to January 2021 and included patients who met the following criteria: HSK diagnosed by cornea specialists, completion of one year of oral VACV treatment, and followed up for a period of at least 12 months after the initiation of oral VACV. The diagnosis of HSK was based on the typical presentation mentioned in HEDS, including stromal keratitis ≥2.5 mm^2^, no epithelial defect longer than 1.0 mm, fewer than 21 cells/field in the anterior chamber, an intraocular pressure less than 30 mmHg, and no clinical signs of a cause other than HSV for the stromal keratitis [4]. The exclusion criteria were patients younger than 18 years of age, co-infected with other infectious pathogens, non-continuous use of VACV, ophthalmic operation within a month of disease onset, and loss of follow-up for more than one year. Recurrence was defined as the typical clinical presentations of HSK with previous episodes on the same eye.

### 2.2. Treatment

We treated the HSK patients based on the HEDS guidelines. Initially, we administered topical steroids and 1 g oral VACV 3 times a day for 7–10 days. On the basis of the clinical responses, oral VACV dosage was reduced to 500 mg daily for a 12-month prophylactic course. The patients were regularly followed up by cornea specialists at the NCKUH, and data pertaining to ophthalmic examinations including slit-lamp images were documented. The patients were divided into recurrence and non-recurrence groups during follow-ups.

### 2.3. Data Collection

We recorded data regarding patient gender, age at disease onset, laterality, follow-up period, recurrence timepoint, systemic diseases, body weight, and height. BMI was divided into 4 classes according to the guidelines of the Bureau of Health Promotion, Department of Health, Taiwan, as follows: underweight—BMI < 18.5 kg/m^2^, normal weight—18.5 ≤ BMI < 24 kg/m^2^, overweight—24 ≤ BMI < 27 kg/m^2^, and obese—BMI ≥ 27 kg/m^2^ [23]. In this study, we analyzed two BMI categories: normal weight (BMI < 24 kg/m^2^) and overweight/obesity (BMI ≥ 24 kg/m^2^).

### 2.4. Statistical Analysis

All statistical analyses were conducted using the Statistical Package for Social Sciences (SPSS) version 22 (IBM Corp., Armonk, NY, USA). The Chi-square test or Fisher exact test was used for testing categorical variables, presented as numbers and percentages. We used the independent two-sample t-test or Mann–Whitney U test for testing continuous variables presented as mean and standard deviation or median and interquartile range (IQR). The recurrence rate was estimated by the Kaplan–Meier method and was presented as a Kaplan–Meier survival curve with a 95% confidence interval (CI). These models were stratified according to the BMI for overweight/obesity category (BMI ≥ 24 kg/m^2^) with the recurrence period as the time scale and presented with hazard ratio (HR) and 95% CI. The Cox proportional hazards models with univariate and forward selection in multivariate analyses were used to identify statistically significant prognostic factors. A two-side *p* value < 0.05 was considered to be significant.

## 3. Results

### 3.1. Patients

**Figure 1:**
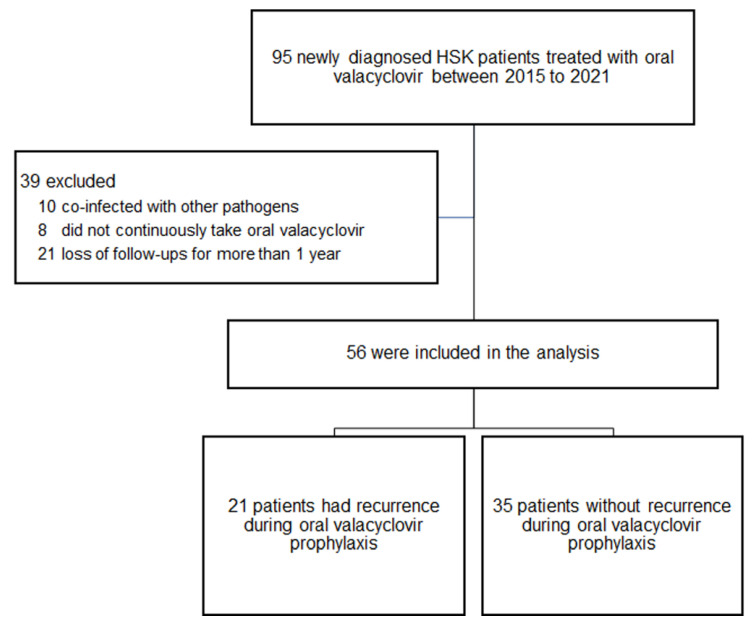
Flow diagram depicting patient selection process for the study.

The details of patient selection are shown in Figure 1. We excluded 10 cases due to co-infection with other pathogens. Of the 85 remaining cases, eight were excluded due to discontinuous intake of VACV, and 21 were excluded due to lack of follow-ups for more than one year. Finally, 56 patients that met the inclusion and exclusion criteria were included in the analysis. The demographic characteristics of the patients are shown in Table 1. A total of 21 patients (21/56, 37.5%) had recurrences (9 males), and 35 did not have recurrences (18 males). The follow-up times were 37.4 ± 15.6 months (IQR, 24.3–51.8 months) and 34.0 ± 16.4 months (IQR, 21.3–44.7 months) in the recurrence and non-recurrence groups (*p* = 0.45), respectively. The age at disease onset was similar between the two groups (*p* = 0.97). Demographics of laterality, hypertension, diabetes mellitus and heart, lung, liver, and kidney diseases were not significantly different between the two groups. Although mean BMI did not show significant difference (*p* = 0.16) between the recurrence (24.5 ± 3.7 kg/m^2^; IQR, 23.3–26.8 kg/m^2^) and non-recurrence groups (22.9 ± 4.4 kg/m^2^; IQR, 20.0–24.0 kg/m^2^), BMI classification reached statistical significance between the two groups (*p* = 0.01). In addition, the recurrence group had significantly more patients with obesity than the non-recurrence group (*p* = 0.01).

### 3.2. HSK Recurrence Analyses

**Figure 2:**
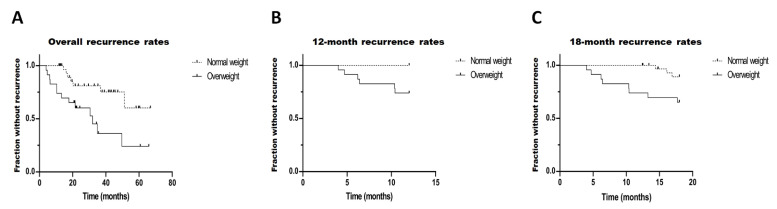
HSK recurrences: (**A**) overall recurrence rates; (**B**) 12-month recurrence rates; (**C**) 18-month recurrence rates.

In the present study, the overall, 12-month, and 18-month recurrence rates were 21/56 (37.5%), 6/56 (10.7%), and 11/56 (19.6%), respectively. Kaplan–Meier analysis showed significant differences between the overweight/obesity and normal-weight patients with regard to the overall, 12-month, and 18-month recurrence rates (log-rank test *p* = 0.006, 0.002, and 0.019, respectively, Figure 2). Using the Cox proportional hazards model (Table 2), we determined that overweight and obesity had a significantly positive relationship with overall recurrence in both univariate (*p* = 0.01) and multivariate analysis (*p* = 0.001). The overweight patients and patients with obesity in our study had a 240% increased risk of recurrence in the univariate analysis (HR, 3.40, 95% CI, 1.34–8.61), and a 428% increased risk in the multivariate analysis (HR, 5.28, 95% CI, 1.91–14.59).

## 4. Discussion

It has been reported that no exposure variables, including psychological stress, systemic infection, exposure to sunlight, menstrual period, contact lens use, and eye injury, are associated with HSK recurrence [12]. In the present study, we found that overweight and obesity were significantly associated with recurrent HSK during oral VACV treatment. This finding might be useful in preventing future HSK recurrences.

Although the mechanisms by which excess body weight is associated with HSK recurrence are not completely understood, we propose a few explanations for the higher recurrence rate in the overweight patients and patients with obesity. First, we surmise that the systemic exposure of the antiviral medications may be lower in the overweight patients and patients with obesity compared to their normal-weight counterparts. Currently, the suggested prophylactic dose for HED recurrence is not adjusted to the body weight. In their study, Turner et al. showed that the intravenous ACV clearance was significantly higher and the maximum serum concentration was significantly lower in the patients with morbid obesity [24]. Moreover, in a previous study, it was shown that the vitreous-to-serum acyclovir concentration was only 25% [25], which means that the exact concentration of ACV is far less in the eye. Therefore, according to our results and those of the above-mentioned previous studies, we suggest that the ideal prophylactic antiviral dosage should be adjusted to body weight [24], especially for the patients with obesity.

The different biological environment of the overweight patients and patients with obesity may be another reason for the higher recurrence rate in these patients. HSK is thought to be an immune response to HSV rather than direct damage [26], and some specific inflammatory platelet-derived transcripts are known to be significantly associated with higher BMI [27]. Among these inflammatory markers, toll-like receptor may be related to HSV recognition and induction of immune response in the cornea [28]. Additionally, in a murine animal model, interleukin-1 and tumor necrosis factor-α were found to be increased in cases of recurrent HSK [26]. These findings suggest that the upregulated inflammatory status of the patients with obesity may contribute to HSK recurrence. Noteworthily, among the 24 obese/overweight patients, 10 (10/24, 41.7%) had diabetes. However, the ratio of diabetes versus obese/overweight was not significantly different between the recurrence (6/14, 42.9%) and non-recurrence (4/10, 40.0%) groups. Although we found that diabetes alone was not significantly correlated with HSK recurrence in this study, the association between obese/overweight with diabetes and HSK recurrence should be evaluated in future studies. Taken together, further studies are needed to evaluate the overall effects of obesity on recurrent HSV keratitis.

To the best of our knowledge, the present study is the first to assess the association between recurrent HSK and excess body weight. The risk of recurrence related to overweight and obesity was revealed to be independent of the other factors in our Cox regression model. However, there are still a few limitations to our study. First, this was a retrospective study, and the sample size was relatively small. However, the advantage of our study was that patients were followed up in a single tertiary medical center and the data were carefully collected. Future prospective studies with larger sample sizes are warranted for better understanding of the relationship between BMI and HSK recurrence. However, despite the lack of clinical trials, it can be suggested on the basis of our findings that the risk of HSK recurrence in overweight patients or patients with obesity might be reduced by losing weight. Second, some patients were unable to cooperate with long-term VACV treatment or receive regular follow-ups in clinical practice. A recent study revealed that compliance to oral antiviral treatment was strongly associated with long-term efficacy of antiviral agents [14]. Therefore, in the present study, to avoid the impact of compliance we included only those patients who were followed up at a single tertiary medical center and completed the 12-month antiviral treatment course. Lastly, although BMI is widely used to assess excess body weight, it does not reflect body fat distribution. Thus, indicators such as waist circumference and waist-to-height ratio may provide more accurate data for the evaluation of the relationship of overweight and obesity with HSK recurrence.

## 5. Conclusions

In conclusion, we found that overweight and obesity are possible risk factors for HSK recurrence during oral VACV prophylaxis. A proper adjustment of the prophylactic dosage for overweight patients and patients with obesity needs to be investigated in the future. Further studies are required to survey the ACV concentrations in the blood and aqueous humour in order to establish an effective prophylactic dosage in recurrent HSK patients.

## Figures and Tables

**Table 1:** Characteristics of the Study Population.

Characteristics	Total (*n* = 56)	Recurrence Group (*n* = 21, 37.5%)	Non-Recurrence Group (*n* = 35, 62.5%)	*p* Value
Gender				0.59
Male	27 (48.2)	9 (42.9)	18 (51.4)	
Follow-up (months)				0.45
Mean ± SD	35.3 ± 16.1	37.4 ± 15.6	34.0 ± 16.4	
Median (IQR)	34.8 (21.6–46.7)	36.5 (24.3–51.8)	33.0 (21.3–44.7)	
Age at disease onset (years old)				0.97
Mean ± SD	63.9 ± 14.2	65.7 ± 8.9	62.9 ± 16.6	
Median (IQR)	66.0 (56.5–72.8)	65.0 (61.5–71.0)	66.0 (53.0–73.0)	
Underlying systemic conditions				
Hypertension	22 (39.3)	6 (28.6)	16 (45.7)	0.26
Diabetes mellitus	15 (26.8)	6 (28.6)	9 (25.7)	1.00
Heart disease	6 (10.7)	1 (4.8)	5 (14.3)	0.39
Lung disease	6 (10.7)	1 (4.8)	5 (14.3)	0.39
Liver disease	2 (3.6)	1 (4.8)	1 (2.9)	1.00
Laterality				1.00
Unilateral	53 (94.6)	20 (95.2)	33 (94.3)	
BMI (kg/m^2^)				0.16
Mean ± SD	23.5 ± 4.2	24.5 ± 3.7	22.9 ± 4.4	
Median (IQR)	23.3 (21.2–26.5)	25.1 (23.3–26.8)	21.9 (20.0–24.0)	
BMI classification				0.01
Underweight	5 (8.9)	2 (9.5)	3 (8.6)	
Normal weight	27 (48.2)	5 (23.8)	22 (62.9)	
Overweight	16 (28.6)	11 (52.4)	5 (14.3)	
Obese	8 (14.3)	3 (14.3)	5 (14.3)	
BMI category				0.01
Overweight/obese	24 (42.9)	14 (66.7)	10 (28.6)	

**Table 2:** Cox Regression Analysis of Variables Associated with Overall Recurrence.

Variables	Univariate Analysis			Multivariate Analysis ^a^			VIF
	HR	95% CI	*p* Value	HR	95% CI	*p* Value	
Overweight/obese	3.40	1.34–8.61	0.01	5.28	1.91–14.59	0.001	1.05
Gender	0.69	0.28–1.66	0.40				
Age at disease onset	1.01	0.98–1.04	0.41	1.03	0.99–1.07	0.12	1.02
Hypertension	0.68	0.26–1.78	0.43				
Diabetes mellitus	1.13	0.43–2.94	0.81				
Heart disease	0.40	0.05–2.99	0.37	0.21	0.03–1.61	0.13	1.04
Lung disease	0.47	0.06–3.55	0.47				
Liver disease	1.02	0.13–7.74	0.99				

CI = confidence interval; HR = hazard ratio; VIF = variance inflation factor; ^a^ Cox regression with forward selection.

## Data Availability

All data generated or analyzed during this study are included in this article. Further enquiries can be directed to the corresponding author.
